# Beyond antibiotics: leveraging microbiome diversity to combat antimicrobial resistance

**DOI:** 10.3389/frmbi.2025.1618175

**Published:** 2025-07-30

**Authors:** Ali Al-Kuwari, Hamad Al-Karbi, Abdulla Al-Khuzaei, Dounia Baroudi, Ghizlane Bendriss

**Affiliations:** Weill Cornell Medicine-Qatar, Qatar Foundation, Education City, Doha, Qatar

**Keywords:** camel milk, antimicrobial resistance (AMR), fermentation, gut microbiota, antibiotics, probiotics, bacteriocins

## Abstract

The best way to fight harmful microbes may not lie in new antibiotics, but rather in leveraging the power of microbes themselves. Antimicrobial resistance (AMR) is a growing global concern, where the overuse of antibiotics has led to the emergence of resistant strains. This paper explores the potential of increasing diversity in gut microbiomes as natural approaches to fight AMR. The promotion microbial diversity is proposed as a promising strategy to reduce dependency on antibiotics by fostering a resilient microbial community. Strategies are discussed to address the loss of diversity caused by antibiotics including diet, probiotics, fecal transplants (FMT) and fermentation of animal/plant products. Preliminary findings from an experiment with camel milk fermentation suggest that fermentation can increase microbial diversity, potentially affecting resistance to common antibiotics such as tetracycline, streptomycin, penicillin, and chloramphenicol, and enhancing microbiome resilience, allowing it to naturally resist pathogens without additional antibiotic use. The results highlight both the benefits and potential risks fermented products. Additionally, FMT, naturally occurring in the animal world, is a promising method to restore microbiome balance and mitigating the impact of AMR. A mechanistic model is discussed to underscore the importance of maintaining microbial balance as an effective strategy for mitigating AMR and promoting long-term health. Further research are needed to better understand the mechanisms behind these changes and their implications for public health. This perspective paper calls for a shift in the approach to AMR, advocating for microbiome-based solutions as a sustainable alternative to traditional pharmaceutical interventions.

## 1 Introduction

Antibiotics are commonly used compounds in modern healthcare that work to fight bacteria. The mechanisms by which they work can include inhibiting cell proliferation by targeting reproduction or altering specific cellular functions (Patel et al., 2023; Wiese-Posselt et al., 2023). Antimicrobial resistance or AMR is defined as the proliferation of a pathogen despite the presence of drugs designed to inhibit its growth (Patel et al., 2023; Wiese-Posselt et al., 2023). The consequence of this development is newfound resistance against existing treatments. Thus, it is more difficult to target and treat AMR. AMR has become a growing concern worldwide; it is projected to cause up to 10 million deaths annually worldwide by 2050 (Tang et al., 2023). Resistance involves genetic changes that allow microbes to grow despite antibiotics, whereas tolerance refers to the ability of a population to survive transient antibiotic exposure without growing, and persistence describes a small subpopulation of dormant cells (persisters) that can survive even high antibiotic doses (Brauner et al., 2016). These processes are closely linked to the host’s microbial ecology and the loss of gut microbial diversity after an antibiotic treatment, disrupts ecological balances that normally suppress resistant and tolerant populations and creates niches where these harmful microbes can emerge and spread. The approach we present in this paper - promoting microbiome diversity through fermented foods and probiotics- mainly targets resistance and tolerance, but may also indirectly target persistence via ecological triggers. Critically, this ecological leverage is missing from most current AMR policies, which tend to focus on genetic resistance alone, overlooking the role of the host microbial environment.

In light of these challenges, microbiome diversity has emerged as a key area of interest. In this perspective paper, we argue that one approach to prevent AMR is via microbial reprogramming enabled by systematic ingestion of fermented foods, especially after antibiotic courses. The fermentation process increases the content and diversity of microorganisms in products, making them interesting food items to modulate gut dysbiosis (Mannaa et al., 2021; Siddiqui et al., 2023).

In addition to their nutritional value, fermented foods have historical and cultural importance across global traditions. From kimchi and kefir to sourdough and lassi, these foods offer a rich landscape for exploring health-supportive microbial interactions. When consumed regularly, these fermented foods promote the development or activity of beneficial gut microbes (Leeuwendaal et al., 2022; Pavlidou et al., 2022).

Camel milk, for example, contains many antimicrobial and antiviral properties due to the presence of hydrogen peroxide, immunoglobulins, lactoferrin, lactoperoxidase, and lysozyme (Swelum et al., 2021; Hamed et al., 2024). It is important to emphasize that camel milk fermentation is presented here as a relevant case study reflective of the regional context in Qatar, not as a singular solution. The goal is to illustrate the broader idea that fermented foods rich in probiotics can meaningfully influence gut microbial ecology and offer natural defenses against resistance to antibiotics (**Table 1**). Other probiotic sources and dietary interventions could and should be investigated, allowing for culturally appropriate and regionally adaptable approaches. Incorporating regionally available fermentation sources not only enhances local relevance but also lowers costs for the consumer and promotes sustainable development. By using what is already culturally familiar and accessible, such interventions can be scaled more effectively, particularly in low- and middle-income countries. We present here in this perspective short article the theory of “fighting microbes with microbes”. To explore the relationship between microbial diversity and AMR, we conducted a series of experiments on raw and fermented camel milk. Results of this first exploration inform on shifts in microbial ecology during fermentation, including reduction in viral and pathogen presence through natural microbial competition and metabolic rebalancing. A model to understand the role of microbial diversity in the gut in AMR will be presented to support our perspective.

## 2 Methods

Fresh camel milk from a local farm was aliquoted into 50 mL tubes labeled R (Raw) and RF (Raw-Fermented), each group had ten replicates (n=10). Using a previously optimized mix of lactic acid bacteria derived from natural camel milk microbiota, enriched through iterative fermentation cycles and selected for acidity resistance (data not shown), bacteria were introduced to the RF tubes, which were then briefly mixed using a vortex, placed on a shaker, and incubated for 20 hours at room temperature to allow for fermentation. To assess acidity tolerance, samples were diluted at 1:10 in sterile phosphate-buffered saline (PBS) adjusted to pH 2.5., and placed into anaerobic tubes and shaking incubator at 37°C for three hours. Serial dilution was performed to a 10^4^ factor in M17 broth, and 200 μL was plated onto M17 agar for overnight incubation 37°C under anaerobic conditions to allow for colony count\ing. A disc diffusion test was used to evaluate antibiotic resistance, a 1:10 dilution was plated onto M17 agar, and antibiotic discs containing tetracycline (30 μg), chloramphenicol (30 μg), penicillin (P10), and streptomycin (S10) were placed on the agar surface. The plates were incubated overnight at 37°C in anaerobic conditions, and the zones of inhibition surrounding the antibiotic discs were measured to assess bacterial resistance to antibiotics. DNA was extracted using the DNeasy PowerFood Microbial Kit (QIAGEN, Cat. No. 21000-100, Hilden, Germany and subjected to shotgun whole metagenomic sequencing allowing detection of bacterial, fungal, and viral components of the microbiota before phylogenetic analysis (CD genomics, NY).

## 3 Results and discussion

### 3.1 Fermentation increases diversity and reduces pathogens

The Krona files attached in supplementary files offer a good visualization of the composition of R and RF. Fermentation allowed an increase in Actinobacteria from 0.1% to 24% of all bacteria, whereas Gammaproteobacteria dropped from 21% to 3% of all bacteria, with Lactobacillales remaining consistent (79% to 73%). Pathogens like *Salmonella* present in the R samples were not retrieved after fermentation (RF). Fermentation led to broader taxonomic representation and compositional shifts, such as an increase in Actinobacteria (from 0.1% to 24%) and the appearance of four distinct *Lactobacillus* species.” Another important observation was the increase in diversity within each group. For example, within Lactobacillales in raw and fermented milk, respectively, fermentation led to greater diversity within the group, including more strains of Lactobacillus, as well as Lactococcus lactis and *Leuconostoc mesenteroides*. However, in raw milk the Lactobacillales population comprised 92% *Lactobacillus* and only 8% of both *Lactococcus lactis* and *Leuconostoc mesenteroides*.

In addition, it contained a higher viral count that ultimately increased the population diversity. Nevertheless, the increase in viruses may be due to the increase in the bacterial reservoir as bacteriophages proliferate in response to increased bacterial hosts; reflecting the microbial diversity and balance, and are not harmful to humans (Vinicius De Melo Pereira et al., 2020). Indeed, an increase of Caudovirales (a large group of bacteriophages) was observed, suggesting that they use bacteria as hosts to survive (Bhatt et al., 2021). Herpesvirales and Salmonella virus IKe were depleted—consistent with a shift in microbial host populations following fermentation.

These preliminary results support the hypothesis of fermentation as a process to increase microbial diversity. The 20-hour timepoint was selected based on prior internal optimization experiments (data not shown), which identified this condition as achieving optimal lactic acid bacteria growth and better resistance to acidity. Prolonged fermentation could increase competition for resources and potentially reduce overall microbial abundance. The increase in diversity can also be explained by multiple factors including improvement of growth conditions and competition for resources (Mannaa et al., 2021), altered pH, and increased microbial interactions (Ilhan et al., 2017). With this new pH, some bacteria thrive, whereas others die, due to the presence of bacteriocin-producing bacteria as well as the production of organic acids, hydrogen peroxide, bioactive peptides, exopolysaccharides, antioxidants, and short-chain fatty acids, all of which support microbial balance and inhibit pathogens (Gao et al., 2019; Leeuwendaal et al., 2022). Overall, fermentation provides a way to not only enhance microbial diversity but also provides health benefits by inhibiting harmful pathogens and improving digestion, making it a valuable natural process in food production.

### 3.2 Fighting microbes with microbes: an ecological model to understand AMR

It seems that when an antibiotic is effective, there is an assumption that it is effective because of the absence of resistance genes in the targeted microbe. However, the biologist knows that microbes interact with each other continuously to inhibit each other’s growth and compete for resources. When an antibiotic works, it is also the result of the entire ecological system present which worked in synergy to create an environment that is inhospitable to its growth. Therefore, it is surprising that the role of the gut ecosystem is so often overlooked when AMR is discussed. Why do strategies and policies focus almost exclusively on genetic resistance and the development of stronger drugs, or fighting antibiotic misuse, but almost never consider that maybe there is something wrong with the host environment? This highlights a critical blind spot—even among those shaping public health policies—probably because of confusions regarding the fundamental biological and ecological processes that underlie the emergence and establishment of resistance.

**Figure 1:**
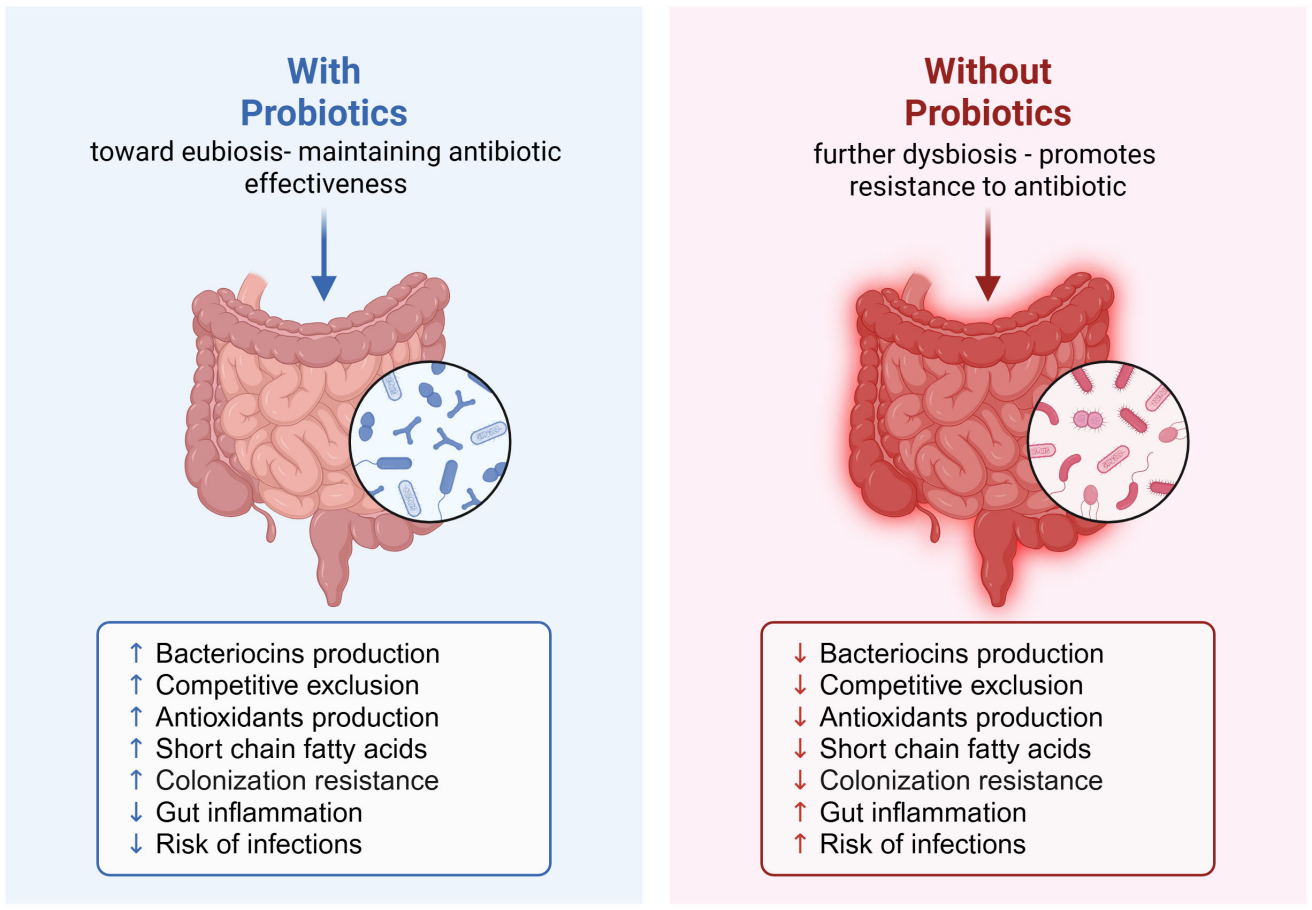
Probiotic-mediated recovery of gut ecology and its role in preventing antibiotic resistance post-antibiotic course. Created in https://BioRender.com.

We therefore would like to present here the mechanistic model where ingesting probiotics during and/or after an antibiotic course could greatly prevent antibiotic resistance (**Figure 1**). In this model, probiotic-mediated recovery of gut ecology involves multiple synergistic mechanisms. Bacteriocins and other antimicrobial peptides such as short chain fatty acids (SCFAs), hydrogen peroxide, antioxidants produced by commensal bacteria target pathogenic competitors and help shape the microbiota composition (Heilbronner et al., 2021). The diversity reinforces the competitive exclusion, which in turn reduces ecological niches available for pathogens by competing for nutrients and adhesion sites (Buffie and Pamer, 2013). In addition, the gut microbiota also acts as an antioxidant system, by mitigating oxidative stress through the activity of certain bacterial taxa, such as *Lactobacillus* (Wu et al., 2022). As it was widely reported in the literature, the SCFAs produced by bacteria such as butyrate, acetate, and propionate, have been shown to strengthen epithelial barrier function, exert anti-inflammatory effects, and modulate immunity (Parada Venegas et al., 2019). By modulating immune responses and through SCFA production, the microbiota contributes to controlling gut inflammation, reducing pro-inflammatory signals and maintaining immune homeostasis (Parada Venegas et al., 2019). Finally, the colonization resistance is reinforced by all these processes, enabling the resident microbiota to prevent the establishment of opportunistic and resistant pathogens (Buffie and Pamer, 2013). Therefore, the restoration of a balanced gut ecosystem decreases the risk of infections, a good example are the microbial interventions to target *Clostridioides difficile* infections (Buffie and Pamer, 2013).

Intrinsic antibiotic resistance refers to a microbe’s innate capacity to resist antibiotics because of its resistance genes (Habboush and Guzman, 2023). In contrast, intrinsic gut-supporting properties refer to the microbe’s characteristics that enhance gut health, such as the production of advantageous compounds or the ability to outcompete harmful microorganisms through productions of bacteriocins (Benameur et al., 2023). Research has shown that the *Lactobacilllus* strains are known for their resistance to ampicillin, erythromycin, gentamicin, streptomycin, tetracycline and vancomycin (Mayrhofer et al., 2010; Anisimova et al., 2022), while Actinobacteria demonstrate resistance to antibiotics such as ceftriaxone, piperacillin/tazobactam, erythromycin, and ciprofloxacin as well as aminoglycosides (Ibrahimi et al., 2020; Urban-Chmiel et al., 2022).

In our pilot exploration, both raw and fermented camel milk exhibited some antibiotic resistance (data not shown). Interestingly, results suggest that the fermentation of the camel milk may lower overall resistance of the product to antibiotics, possibly due to increased microbial diversity (**Table 1**). We hypothesize that resistance in lactic acid bacteria may allow these strains to survive antibiotic exposure and continue producing beneficial metabolites such as short-chain fatty acids (SCFAs), which are essential for gut barrier integrity and immune function (Blaak et al., 2020; Fusco et al., 2023; Zhang et al., 2023).

**Table 1:** Comparative overview of raw and fermented camel milk microbiota after 16sRNA sequencing.

	Raw Camel Milk	Fermented Camel Milk
Total Microbial Composition	99% Bacteria 1% Viruses	74% Bacteria 26% Viruses
Bacterial Composition	79% Lactobacillales • 92% *Lactococcus lactis* • 7% *Leuconostoc mesenteroides* 21% Gammaproteobacteria	73% Lactobacillales • 37% *Lactococcus lactis* • 29% *Lactobacillus acidophilus* *• 17% Leuconostoc mesenteroides* *• 12% Lactobacillus plantarum* • 3% *Lactobacillus delbrueckii* 24% Actinobacteria 3% Gammaproteobacteria
Viral Composition	88% Caudovirals 6% Herpesvirales 5% Salmonella virus IKe	~99.99% Caudovirilas

This observation leads to an important consideration for future interventions: could probiotics be administered simultaneously with antibiotics to preserve microbial resilience? While still speculative, this direction opens the door for comparative studies to examine the effectiveness in increasing gut diversity by co-ingestion or post-ingestion of probiotics versus spontaneous repopulation after an antibiotic course. This is an area currently overlooked in public health policy, and research in this direction could support new approaches to how we can keep current antibiotics working.

Nevertheless, there remains a valid concern regarding the risk of horizontal gene transfer, where antibiotic resistance genes may be shared between commensals and pathogens during fermentation or *in vivo* (Michaelis and Grohmann, 2023; Muteeb et al., 2023). While this presents a potential risk, it can be mitigated through careful strain selection, gene editing, CRISPR-Cas9 systems, and phage therapy (Emamalipour et al., 2020; Muteeb et al., 2023). These tools offer modern solutions to ancient microbial dynamics and could allow us to harness the benefits of probiotics while minimizing associated risks.

## 4 Conclusion

When an enriched and balanced gut environment (eubiosis) is maintained, it less likely for resistant strains to dominate. If this diversity is not restored post-antibiotic use, the result is often dysbiosis. In such a depleted microbial landscape, surviving resistant strains can thrive unchecked, setting the stage for poor responses to future antibiotic treatments.

Antibiotic resistance does not result from a genetic battle but also from an ecological imbalance. Protecting the gut microbiome through strategies such as co-administering probiotics could represent a transformative step in preserving antibiotic efficacy. A paradigm shift toward ecosystem-centered policies is urgently needed.

## Data Availability

The datasets presented in this study can be found in online repositories. The names of the repository/repositories and accession number(s) can be found in the article/**Supplementary Material**.
