# Supplementary material for: Beyond antibiotics: leveraging microbiome diversity to combat antimicrobial resistance
Source: Front Microbiomes. 2025 Jul 30;4:1618175. doi: 10.3389/frmbi.2025.1618175 (PMC12993663; doi:10.3389/frmbi.2025.1618175)
Supplement: Supplementary file 1 [file Supplementaryfile1.docx]

**Interactive KRONA visualizations** of microbial composition are available to download via the following link. These dynamic HTML files allow zooming and exploration of taxonomic hierarchies and were used to compare raw (R) and fermented (RF) camel milk samples.

<https://drive.google.com/drive/folders/1RvfX4MmLWuip57h0me_8fQEr09iu7eB9?usp=sharing>
